# Distribution and Abundance of MAAs in 33 Species of Microalgae across 13 Classes

**DOI:** 10.3390/md8041273

**Published:** 2010-04-16

**Authors:** Carole Anne Llewellyn, Ruth Louise Airs

**Affiliations:** Plymouth Marine Laboratory, Prospect Place, The Hoe, Plymouth PL1 3DH, UK; E-Mail: ruai@pml.ac.uk

**Keywords:** MAAs, microalgal cultures, phytoplankton

## Abstract

We provide a direct comparison of the distribution and abundance of mycosporine-like amino acids (MAAs) in a diverse range of microalgal cultures (33 species across 13 classes) grown without supplementary ultraviolet radiation (UV). We compare the MAAs in cultures with those present in characterised natural phytoplankton populations from the English Channel. We detected 25 UV absorbing compounds including at least two with multiple absorption maxima. We used LC-MS to provide chemical characterisation of the six most commonly occurring MAAs, namely, palythene, palythine, mycosporine-glycine, palythenic acid, porphyra-334 and shinorine. MAAs were abundant (up to 7 pg MAA cell^−1^) in 10 species, with more minor and often unknown MAAs in a further 11 cultures. Shinorine was the most frequently occurring and abundant MAA (up to 6.5 pg cell^−1^) and was present in all but two of the MAA-containing species. The study provides further insight into the diversity and abundance of MAAs important from an ecological perspective and as potential source of natural alternatives to synthetic sunscreens.

## 1. Introduction

Mycosporine-like amino acids (MAAs) are a group of over 20 ultraviolet (UV) absorbing compounds present in a diverse range of aquatic organisms where they act as sunscreens to reduce UV-induced damage. Whilst their main role is in screening against energetic UV radiation, MAAs also play a role in protecting against sunlight damage by acting as antioxidant molecules scavenging toxic oxygen radicals [1,2]. Further roles include acting as compatible solutes to protect cells against salt stress where they are involved in protection against desiccation or thermal stress in certain organisms and as intracellular nitrogen reserves [2].

Mycosporines were first identified in fungi as having a role in UV-induced sporulation [3]. Their relatives the MAAs have since been found in a diverse variety of freshwater and marine organisms (more than 380 marine species) including cyanobacteria, macro- and microalgae, corals as well as many marine invertebrates such as sea anemones, limpets, shrimp, sea urchins and vertebrates including fish and fish eggs [4,5]. Geographically, MAAs are distributed ubiquitously, occurring in tropical, temperate and polar aquatic environments [4–6]. Intracellularly, MAAs are found distributed in the cytoplasm of cells although it has also been suggested that they can be released extracellularly into colonial mucilage in *Phaeocytis* sp. providing enhanced UV protection to themselves and possibly to other community members [7].

**Figure 1:**
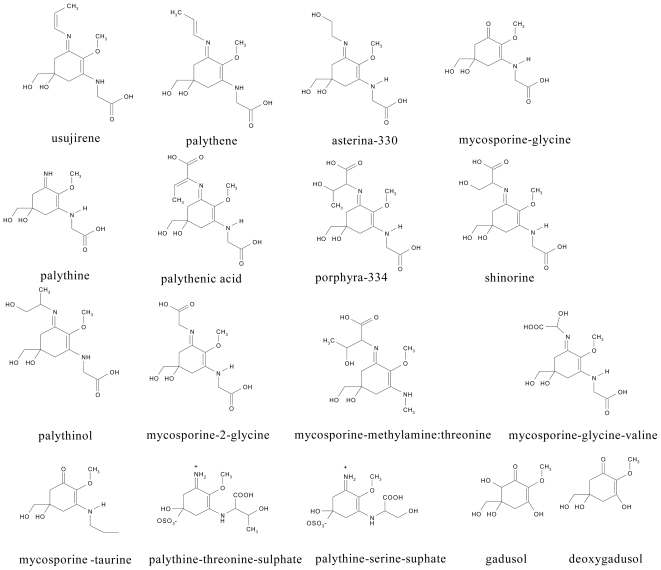
Chemical structures of MAAs referred to in the text.

As a class of compounds, MAAs are polar (water soluble) with low molecular weights (<400 Da) and have a chemical structure based on either a cyclohexenone or cyclohexenimine ring structure with amino acid substituents (Figure 1). The conjugated double bonds within the molecule result in broad band absorptions with wavelength maxima (λ_max_) ranging from 310 nm in the ultraviolet-B for cyclohexenone based structures (UV-B, 280–320 nm) to 360 nm in the ultraviolet-A (UV-A, 320–400 nm) for cyclohexenimine based structures [8]. Changes in the λ_max_ are caused by substituent effects with organisms generally containing a variety of MAAs protecting across a wide UV band range.

Evidence suggests that most MAAs in autotrophic eukaryotic and prokaryotic organisms are biosynthesised *de novo via* the shikimate pathway [9–11]. Higher organisms were until recently believed to lack the shikimate pathway and assumed to accumulate MAAs from their diet [12]. Recent evidence suggests that genes encoding for shikimate biosynthesis are indeed present in some higher organisms [13,14]. The large diversity of mycosporine molecules found in nature is suggested to be produced by variations in late biosynthetic steps, which add free amino-moieties to the core structure [11]. To date, at least 19 structurally distinct MAAs have been recognized [5,15]. More unusual MAAs include those containing sulphate esters as reported to occur in the coral *Stylophora pistallata* [16] and those where MAAs are covalently linked to oligosaccharides through the imine substituents as reported in the terrestrial cyanobacterium *Nostoc commune* [17]. Lastly, in addition to several novel MAAs present in 3 different species of the dinoflagellate *Alexandrium* spp. atypical compounds containing two main absorption bands were present and were tentatively characterised as esters formed by condensation of two MAAs [18]. Additional studies reveal further novel and unidentified MAA-like compounds [19–24].

The role MAAs have in functioning as Nature’s sunscreen compounds has led to them being widely studied from an ecological perspective and their importance with regard to biological, chemical and physiological aspects has been the subject of several reviews [5,12,15]. A database referencing studies on MAAs in cyanobacteria, micro- and macroalgae from diverse habitats has been compiled [25] and updated [26], and is available at www.biologie.uni-erlangen.de/botanik1/html/eng/maa_database.htm.

Whilst it is evident that MAAs are present in a wide number of species (>30 marine and freshwater species) most studies focus on only a few algal species and there have been no comprehensive comparative surveys of MAAs across or within classes within one study using the same methodology. Comparing results from various published studies, whilst invaluable, is made difficult because of the wide range of methodologies used in the separation of MAAs and because most studies do not provide information on the concentrations of MAAs.

From a more applied industrial biotechnology perspective MAAs are becoming important as compounds of interest to supplement and/or replace commercially available sunscreens, particularly as the requirement for natural products and replacement of petrochemical based products is growing. From an applied perspective the broad band absorption of the MAAs particularly in the UV-A and their high UV absorption coefficients are attractive properties in the search for synthetic replacements [27].

Here we report the identification, distribution and concentration of MAAs in 33 microalgal cultures. The cultures were selected to provide a representative analysis across 13 classes and to provide focus on species previously reported to contain MAAs. The cultures consisted of eleven dinoflagellates, six cryptophytes, two diatoms, two chlorophytes, two chrysophytes, three prymnesiophytes, and one of each from the chlorarachniophytes, cyanophytes, euglenophytes, eustigmatophytes, prasinophytes raphidophytes and rhodophytes (Table 1). All but two of the cultures were marine species: *Nostoc commune* is a terrestrial cyanobacterium and *Euglena gracilis* is a freshwater species. All but one of the cultures were autotrophic; *Oxyrrhis marina* is a heterotrophic dinoflagellate which was grown with a diatom. Cultures were grown under fluorescent “daylight” without supplementary UV. Many of the species we examined in culture are known to be present at our sampling site in the English Channel (www.pml.ac.uk/L4); a comparison was therefore made with natural populations of phytoplankton sampled from the English Channel during July and August when the abundance of dinoflagellates and diatoms is known to be high. Our objective here was to match up taxonomic information to determine possible sources of MAAs in the natural environment. Our results give improved insight into the diversity and abundance of MAAs, important from an ecological perspective and as a guide for industrial biotechnologists.

## 2. Results and Discussion

### 2.1. Chromatographic separation and detection of MAAs

**Figure 2:**
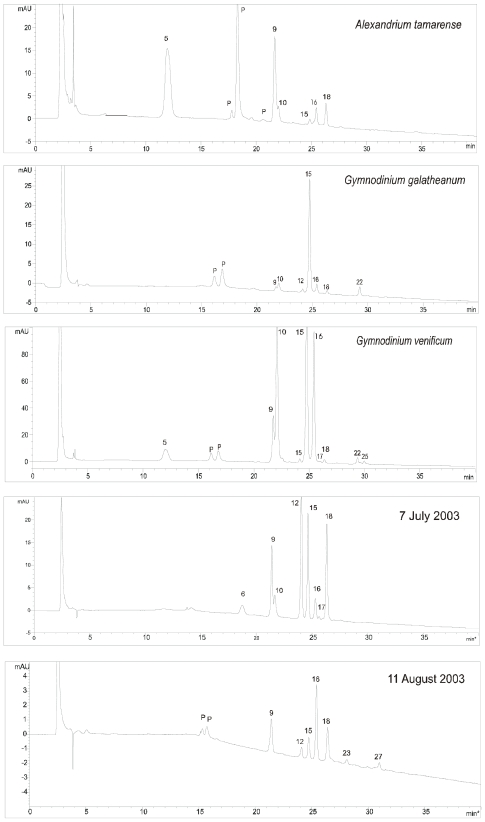
HPLC chromatograms (330 nm) of representative cultures and phytoplankton samples. See Table 1 for peak assignments. P = photosynthetic pigment.

MAAs were analysed from 33 species of microalgae using anion exchange high performance liquid chromatography (HPLC) with photodiode array (PDA) detection (Figure 2; Table 1). The order of elution of the individual MAAs was reversed compared to that of reversed phase (RP) chromatography. For example, mycosporine-glycine (9), porphyra-334 (16) and shinorine (18) were strongly retained using anion-exchange (*t*_R_ = 21.8, 25.4 and 26.3 min respectively) but elute relatively early under RP conditions and conversely, palythene (5) one of the first MAAs to elute using anion-exchange chromatography exhibits a long retention time under RP conditions [18,28]. We detected 25 MAA or MAA-like compounds across 33 algal species, including usijerene (4), palythene (5), palythine (10), mycosporine-glycine (9), palythenic acid (15), porphyra-334 (16) and shinorine (18). Two peaks (1, 2) were characterised with multiple absorption maxima (Table 1). These peaks were symmetrical and determined as pure using peak purity software. In addition we detected four compounds with λ_max_ between 276 and 294 nm (peaks 3, 7, 11, 13; Table 1).

### 2.2. Distribution and abundance of MAAs in cultures

The distribution and abundance of the MAAs in the cultures examined are shown in Table 1. MAAs were detected in 20 out of 33 species examined and occurred most frequently and at highest concentrations in the dinoflagellates (Table 1). MAAs occurred in all but two of the dinoflagellates (*Amphidinium carterae* and *Heterocapsa triquetra*) examined. MAAs have previously been reported in *A. carterae* and *H. triquetra* in cultures grown with enhanced UV indicating that these species may require UV to induce MAAs [29–31]. The widespread occurrence of MAAs in dinoflagellates has been previously reported in a spectrophotometric survey that found surface bloom forming species and dinoflagellates, in particular, have the capacity to accumulate high amounts and a diversity of UV absorbing compounds [22]. In our study *Glenodinuim foliaceum* and *Scrippsiella trochoidea* had the highest MAA concentrations of all the cultures examined at >6000 fg cell^−1^ and *Gymnodinium galatheanum*, and *Gymnodinium venificum* had concentrations of >1500 fg cell^−1^ (Table 1). These concentrations are consistent with those reported in the literature in cells not exposed to UV [18,32] but are considerably lower in concentration than those reported for UV exposed cells where concentrations of >300 pg cell^−1^ have been measured in *Alexandrium tamarense*. [33].

The heterotrophic dinoflagellate *Oxyrrhis marina* (containing intracellular diatoms) is believed to be unable to synthesise MAAs, although we found it to contain MAAs at similar concentrations to the other dinoflagellates and exceeding those of the diatom cultures analysed. The MAA composition of *Oxyrrhis marina* was dominated by high levels of shinorine, which was also observed for other dinoflagellates analysed (Table 1). This suggests that the MAAs in *O. marina* did not originate from the diatom food source and were possibly synthesised. Specific inhibitors of the shikimic acid pathway block accumulation of MAAs suggesting MAA biosynthesis in autotrophic eukaryotic and prokaryotic organisms is derived from a shikimate branch point [9–11]. Higher organisms reputedly lack the central pathway to synthesize shikimate-derived ‘essential’ aromatic amino acids [34], although genes encoding shikimic acid pathway enzymes have recently been found in a sea anemone [14]. Notably, *O. marina* contains a paralogue of the shikimate biosynthetic enzyme AroB, obtained by horizontal gene transfer from a cyanobacterial source [13]. The implications of this need to be further explored, and will be assisted by studies such as [35] which used a bioinformatics approach to propose gene products involved in the biosynthesis of the MAA precursor, deoxygadusol.

Of the three species of prymnesiophytes examined, *Isochrysis galbana* and *Emiliana huxleyi* contained MAAs at low concentrations and *Phaeocystis globosa* contained no detectable MAAs. This contrasts with other studies where cultures of the colonial form of *Phaeocystis antarctica* and natural populations of *Phaeocystis pouchetii* were shown to contain UV absorbing compounds although levels were highest under UV or high PAR irradiance conditions [7,36–39]. Notably, the single celled flagellate phase of *P. pouchetti,* which is absent in the acellular matrix of the colonial form, also lacked UV absorbing compounds [7].

Of the two species of diatoms examined, *Thalassiosira weissflogii,* a centric species, contained low concentrations of porphyra-334 whereas the pennate diatom *Phaeodactylum tricornutum* contained no detectable MAAs. This is consistent with a previous study [40] that found centric species of Antarctic diatoms contained higher concentrations of MAAs than pennate species. Only one of the six species of cryptophytes, *Rhodomonas baltica*, contained UV absorbing compounds, with high levels of a compound giving λ_max_ at 310 nm. The species analysed from chlorarachniophytes, *Chlorarachnion reptans*, contained only shinorine whilst the raphidophyte, *Heterosigma akashiwo* was dominated by asterina-330. Species analysed within the chlorophytes, eustigmatophytes, prasinophytes and rhodophytes lacked commonly occurring MAAs although several species contained minor amounts of unknown UV-absorbing compounds (Table 1). The absence or low levels of MAAs in the chlorophytes, eustigmatophytes, prasinophytes and rhodophytes is consistent with few studies having reported MAAs in these classes and consistent with the spectrophotometric UV screening study undertaken by [22]. More recently MAAs designated as 322nm-MAA and 324nm-MAA were identified within a distinct clade of the Trebouxiophycea of the Chlorophyta [41].

### 2.3. Characteristics of MAAs in microalgal cultures

There were six frequently occurring and abundant MAAs in the microalgal cultures examined: palythene (peak 5), mycosporine-glycine (peak 9), palythine (peak 10), palythenic acid (peak 15), porphyra-334 (peak 16) and shinorine (peak 18). Shinorine (18) was the most abundant and frequently occurring MAA and occurred in all MAA-containing cultures except for the raphidophyte *Heterosigma akashiwo* and the terrestrial cyanobacterium *Nostoc commune* (Table 1). A diverse suite of MAAs including mycosporine-glycine, palythine, porphyra-334, palythenic acid and palythene were present in the dinoflagellate *Gymnodinium venificum*, each MAA at high concentration (>100 fg cell^−1^); shinorine was also present but at low concentration (10 fg cell^−1^; Table 1). High concentrations of MAAs were present in other dinoflagellates, exceptionally high concentrations of shinorine (6497 fg cell^−1^) were present in *Glenodinium foliaceum* and high amounts of palythenic acid (1273 fg cell^−1^) were present in *Gymnodinium galatheanum* (Table 1). Whilst *H. akashiwo* did not contain shinorine it was the only culture examined containing asterina-330 (peak 8).

In addition to the frequently occurring MAAs, many cultures contained less well documented and more minor MAAs. Peak 14 eluting just before palythenic acid with a λ_max_ at 310 nm was found in high concentrations in the dinoflagellate *Scrippsiella trochoidea* (28 fg cell^−1^) and in the rhodophyte *Rhodomonas baltica* (140 fg cell^−1^). The λ_max_ at 310 nm is consistent with a cyclohexenone structure indicative of mycosporine-taurine, mycosporine-serine or mycosporine-glutamine. Peak 20 eluted after shinorine, gave a λ_max_ at 320 nm and was present in the terrestrial cyanobacterium *Nostoc commune*, in four dinoflagellates and all three species of *Prorocentrum* analysed. The spectral and elution properties of this compound are consistent with an MAA absent in glycine at the C3 position of the molecule e.g., palythine-threonine or palythine-serine. Notably, a polar peak with a λ_max_ at 320 nm has previously been observed in chromatograms of extracts from *Alexandrium* sp [18]. We also observed two novel MAA-like compounds (peaks 12 & 19) with distinct and previously unreported absorption maxima: Peak 12 (λ_max_ 342 nm) was present in the dinoflagellates *Gymnodinium galatheanum* and *Gymnodinium venificum*. Peak 19 (λ_max_ 352 nm) was present in the dinoflagellate *Prorocentrum micans*. There were minor peaks eluting after shinorine with λ_max_ at 334 and 334/336 nm (peaks 22, 25–27) in many cultures. The spectral and elution properties of these peaks are consistent with compounds such as mycosporine-2 glycine and mycosporine-glycine valine.

Compounds with λ_max_ above 360 nm (*i.e.*, above the λ_max_ normally associated with MAAs), were found in three species although at relatively low concentration. Peak 24 with λ_max_ at 384 nm was found in the Euglenophyte *Euglena gracilis* and peak 29 with λ_max_ at 374 nm was found in the raphidophyte *Heterosigma akashiwo* and in the rhodophyte *Porphyridium purpureum*. Similar compounds with λ_max_ > 360 nm have been observed in other studies with compounds absorbing between 370 and 380 nm in the dinoflagellate *Gymnodinium catenatum* [18,22], and compounds with λ_max_ at 380 and 368 nm in phytoplankton populations from the Icelandic Basin and the English Channel [38,42].

Compounds with more than one UV absorption band were found in two cultures. The chromatograms of MAA extracts from the terrestrial cyanobacterium *Nostoc commune*, and the heterotrophic dinoflagellate *Oxyrrhis marina* exhibited prominent early eluting peaks that exhibited three absorption bands (Table 1). These compounds may be related to sunscreen compounds with multiple λ_max_ identified in *Nostoc commune* and found to consist of MAAs covalently linked to oligosaccharides [17].

All cultures including those absent in MAAs, exhibited components (peaks 3, 7, 11 and 13) with λ_max_ ranging between 270 and 296 nm. The most abundant and frequently occurring, peak 13, had a λ_max_ at 294 nm corresponding to that of the MAA precursor, gadusol. There have been suggestions of a biological relationship between MAAs, 6-deoxygadusol (λ_max_ 268 nm) and gadusol. However, gadusols have not been reported to occur in microalgae and further detailed chemical structural analysis using mass spectrometry and nuclear magnetic resonance is required to identify these compounds. In addition to the main absorption band maximum at 276 nm for peak 11, the four cultures *Glenodinium foliaceum*, *Oxyrrhis marina*, *Nannochloropsis oculata* and *Rhodomonas baltica* contained additional absorption maxima for this peak between 278 and 294 nm, and between 310 and 334 nm. Likewise for peak 13 (λ_max_ 294 nm), an additional maximum was observed in *Prorocentrum lima* at 336 nm.

### 2.4. Factors influencing induction and distribution of MAAs

The intensity and spectral distribution of radiation has been shown to influence the induction of MAAs. In a study on *Gymnodinium sanguineum* grown under cool-white fluorescent light (without supplementary UV), MAA levels were about 14 fold higher relative to chl-a in cultures grown under high light (76 W m^−2^) compared to those under low light (15 W m^−2^) [43]. Blue light and UV-A have been shown to interact to boost the synthesis of the MAA shinorine in the macroalgae *Chondrus crispus* [44] and UV-A irradiance has been shown to be more important for the induction of shinorine and palythine than UV-B [45]. Blue light and UV-A have also been reported to induce the synthesis of MAAs in diatoms whereas UV-B was required for induction of MAAs in *Phaeocystis antarctica* [36]. UV-B was also found to increase levels of MAAs in the dinoflagellates *Alexandrium tamarense* and *Heterocapsa triquetra* [31]. Another study [46] investigating the induction of MAAs in 18 species of Antarctic red macroalgae found no consistent MAA induction patterns even for individual MAAs. Furthermore intra-strain and phylotype differences have been observed for dinoflagellates *Alexandrium tamarense* and symbiotic *Symbiodinium* spp. respectively [31,47]. Natural populations of phytoplankton are therefore likely to contain differing compliments of MAAs varying according to prevailing solar irradiance spectral distribution. In our study it is possible that the fluorescent lights used to irradiate the microalgal cultures may have favoured the production of MAAs absorbing in the near PAR (for example shinorine), compared to UV-B absorbing MAAs (for example mycosporine-glycine).

On the other hand the occurrence of MAAs in cultures grown without supplementary UV radiation suggests that factors other than UV exposure may also be important in regulating MAA synthesis. Although irradiance is the main driving factor in the production of MAAs, nutrient availability, salinity and temperature are also known to affect accumulation [2,5]. A source of nitrogen is essential for the synthesis of MAAs. High concentrations of ammonium (300 μM) have been shown to significantly increase MAA concentrations in the red macroalgae, *Porphyra* [48] and nitrate limitation has been found to reduce concentrations of MAAs [49]. Likewise phosphate depletion was found to increase concentrations of shinorine, palythine and asterina-330 in cultures of the dinoflagellate *Glenodinium foliaceum* [50]. What is emerging is that the induction, formation and accumulation of individual MAAs is a highly flexible and species and even intra-strain specific mechanism.

### 2.5. Comparison with natural samples

Many of the species we examined in culture are known to be present in the English Channel during our chosen sampling periods. Phytoplankton sampled from the English Channel in July 2003 revealed high concentrations of MAAs (173 ng L^−1^), and a wide diversity of compounds (Table 1). In particular there were high concentrations of a UV-absorbing compound (32 ng L^−1^) exhibiting a λ_max_ at 342 nm (peak 12, Figure 1), which does not correspond to any MAA reported to date. This compound (peak 12) was also present in cultures of *Gymnodinium galatheanum* and *Gymnodinium venificum*. On referring to the L4 database (www.pml.ac.uk/L4) we found that in July high numbers of dinoflagellates (64.6 mg C m^−3^), comprising almost exclusively *Gyrodinium aureolum* (62.6 mg C m^−3^), dominated the phytoplankton biomass (104.5 mg C m^−3^). Co-occurring with peak 12 in July were high concentrations of mycosporine-glycine (9), palythenic acid (15) and porphyra-334 (16) (Table 1). These MAAs were also abundant in cultures of *G. galatheanum* and *G. venificum* (Table 1). Our results suggest that the novel UV-absorbing compound (12) may be unique to *Gymnodinium* and *Gyrodinium* spp. Notably, *Gymnodinium* and *Gyrodinium spp*., both from the family Gymnodiniaceae (order: Gymnodiniales), are unique among the dinoflagellates in that they do not contain the characteristic pigment peridinin, but rather fucoxanthin-alkyl esters [51]. Peak 6 (λ_max_ 314 nm), present in the July sample was also present in cultures of the prasinophyte *Nephroselmis rotunda* and the rhodophyte *Porphridium purpureum*, although neither of these species were identified in the L4 database.

In August 2003 concentrations of MAAs were lower than in July (59 ng L^−1^) and although the MAAs present were similar the relative abundance was different (Table 1). Concentrations of peak 12 decreased (3.5 ng L^−1^; Figure 1) and porphyra-334 became more prominent (15 ng L^−1^). This was consistent with the phytoplankton identified: although *G. aureolum* (33 mg C m^−3^) continued to dominate, the phytoplankton biomass (61.2 mg C m^−3^) was lower than in July and there were also other dinoflagellates present. There were two additional UV absorbing compounds (peaks 23 and 27) present in August. Peak 23 was found in the culture of *Prorocentrum minimum* and in the L4 phytoplankton. Peak 27 was found in cultures of *Prorocentrum micans* and in *Nannochloropsis oculata* although neither of these species were identified in the phytoplankton. It is possible that peak 27 also originated from *P. minimum* as the chromatogram of the culture extract did show small unidentifiable peaks eluting after peak 26. It is also possible that peak 27 originated from a species not examined in our cultures. Such differences could also be attributed to the natural populations being exposed to natural levels of UV irradiance.

### 2.6. Spectrophotometric analysis of cultures

Spectrophotometric analysis of extracts for detection of MAA absorbance should be approached with caution as extracts can contain absorbance in the UV resulting from photosynthetic pigment derivatives. Parallel spectrophotometric analyses of MAA extracts revealed all cultures to contain absorbance maxima between 330 and 340 nm although 12 of these cultures did not contain detectable MAAs using HPLC. A similar result was reported previously [22]: an extract of the diatom *Chaetoceros affinis* contained UV absorbance when analysed spectrophotometrically but contained no detectable MAAs when analysed by HPLC. Further analysis of our extracts using HPLC pigment methodology revealed the UV absorbance was contributed to by the photosynthetic pigment derivatives chlorophyllide-a, containing a shoulder absorption with λ_max_ at 338 nm and cis-fucoxanthin containing an absorption band with λ_max_ at 334 nm.

## 3. Experimental

### 3.1. Culturing and sampling

Cultures were grown in 200 mL Erlenmeyer flasks in appropriate media (Table 2) and illuminated at an irradiance of 98 to 146 μmol m^−2^ s^−1^ with “daylight” fluorescent tubes (Phillips) on a 12:12 hour light-dark cycle. No supplementary UV radiation was provided. Cultures were maintained at 15 °C, mixed by swirling daily and sampled during exponential growth phase. Prior to analysis, 50 mL aliquots of culture were centrifuged to produce an algal pellet and then freeze dried overnight. Cell concentrations were determined from the number of cells harvested. Cell counts were determined from an aliquot of culture (1 mL) which was stained and preserved in 2% Lugol′s iodine and settled in sedimentation chambers (either a Segdwick Rafter chamber or a Fuchs-Rosenthal blood counting chamber, depending on cell size) using an inverted light microscope.

For comparison particulate samples were collected during July and August 2003 from surface seawater from a long term monitoring station in the western English Channel. Details of this monitoring station (L4) together with results from weekly analysis of phytoplankton can be found at www.pml.ac.uk/L4. July and August were chosen as sampling times for their high likely abundance of diatoms and dinoflagellates with high levels of MAAs as shown from the L4 database (www.pml.ac.uk/L4) and as previously observed in a seasonal study [38]. Particulates were filtered onto 25 mm GF/F filters by vacuum filtering one litre volumes of seawater. Filters were stored frozen in liquid nitrogen until analysis.

### 3.2. Extraction and HPLC analysis

MAAs were extracted from the algae and seawater particulates with an ultrasonic probe (30 s, 50 W) using 1 or 2 mL 75% acetonitrile and then centrifuged (4,000 rpm for 5 min) to remove cellular debris. Prior to HPLC, extracts were analysed using a dual beam spectrophotometer (Cecil) scanning between 200 and 650 nm. For HPLC analysis the extracts were injected (100 μL) onto a system comprising an autosampler, quaternary pumping system and photodiode array detector operated using ChemStation software (Agilent 1100 series). MAAs (and other UV absorbing compounds) were separated using a Luna NH_2_ column (Phenomenex; 250 × 4.6 mm; 5 μm particle size) and gradient elution. Solvents (Fisher) and ammonium carbonate (Sigma-Aldrich Ltd.) were HPLC grade. Solvent A = 85% acetonitrile: 15% 0.1M ammonium carbonate buffered to pH 10 and solvent B = 75% 0.1M ammonium carbonate: 25% acetonitrile. To avoid solvent outgassing we premixed A and B and degassed them by gently bubbling with N_2_ gas for 10 min. The solvent composition was programmed as follows: 100% A (10 min), linear gradient to 25% A, 75% B over 35 min. The flow rate was 1 mL min^−1^. Chromatograms were recorded at 330 and 310 nm and spectra were collected between 250 and 500 nm.

For compatibility with HPLC solvents, MAAs were extracted into 75% acetonitrile. We found that 75% acetonitrile was as efficient at extracting MAAs as 25% and 100% methanol (data not shown). Increasing the length of extraction time or temperature did not increase the amounts of MAAs extracted and ultrasonication with a probe extracted identical amounts of MAAs as achieved using homogenisation. We did however find that extraction was less efficient if samples were not frozen or freeze dried prior to extraction. Extraction efficiency, determined by re-extraction using both methanol and acetonitrile, was >95%.

### 3.3. MAA identification and quantification

Eight abundant MAAs were isolated from cultures using preparative HPLC. These isolated MAAs were analysed by liquid chromatography/tandem mass spectrometry using a Dionex LC connected to ThermoFinnigan LCQ ion trap mass spectrometer *via* an ESI interface. LC/MS conditions were as follows: Capillary temperature 200 °C; ion time 5 ms; Sheath gas flow 60 (arb. Units); Auxillary gas flow 20 (arb. Units); Source voltage 4.5 kV; Source current 80 μA; Capillary voltage 22 V; LC conditions were as described above, with ammonium carbonate substituted by ammonium solution (pH 10.2). LC/MS^n^ was used to check the purity of the isolated MAAs as well as obtain the mass to charge ratio of the protonated molecule and fragmentation spectra to MS^3^ for each MAA. MAAs were assigned as usijerene, palythene, asterina-330, mycosporine-glycine, palythine, palythenic acid, porphyra-334 and shinorine based on on-line UV/vis spectra, molecular mass derived from the mass to charge ratio of the protonated molecule and fragmentation data [52]. Response factors (ng/HPLC peak area) were determined for the isolated MAAs in triplicate by solubilising each MAA in water, obtaining a spectrophotometric scan, calculating concentration using published extinction coefficients [15] and then reinjecting a known concentration onto the HPLC column. Three injection peak areas for each standard were averaged and gave a relative standard deviation of <1%. Where a response factor for an individual MAA was not available (e.g., Porphyra-334), the RF of the component with the closest UV/vis absorption spectrum was used (*i.e.*, shinorine in this case) to provide a best estimate of concentration.

## 4. Conclusions

Our comprehensive study of MAAs in 33 species of microalgae across 13 classes contributes to our growing knowledge on the diversity and abundance of mycosporine-like amino acids in algae and natural phytoplankton populations. Whilst there have been a number of studies assessing MAAs in microalgae there have been few to assess and quantify such a wide number of species in one study using the same methodology. Futhermore, our study confirms the importance of using HPLC to separate MAAs rather than using spectrophotometry which can produce ambiguous results due to interference from photosynthetic pigment derivatives. It would be useful to repeat our study using the same strains grown with and without UV-A and UV-B. From this we could determine if those strains lacking MAAs without UV are able to induce them with UV and also how levels of induction compare across all strains. Furthermore the effect of nutrients on the induction of MAAs especially in combination with UV needs further study. This would lead to a better understanding of species specific and indeed intra-strain and phylotype differences. More research is required on chemically characterising the many MAA-like compounds using analytical techniques such as tandem LC-MS and NMR. In this study, for example, there were a number of unidentified peaks eluting late in our chromatograms with absorption maxima matching that or similar to that of porphyra-334. In addition, the cultures of *Gyrodinium* and *Gymnodiniun* spp. revealed the presence of a novel UV absorbing compound λ_max_ 342 nm (peak 12), and this novel UV absorbing compound was also found in abundance in surface water phytoplankton from the English Channel. Continuing research on the diversity and abundance of MAAs is necessary to obtain a better understanding of UV protection in phytoplankton from an ecological perspective and as potential to replace or complement current synthetically derived sunscreen compounds.

## Figures and Tables

**Table 1:** Concentrations of MAAs in microalgal cultures (fg cell^−1^) and in English Channel natural phytoplankton populations (ng L^−1^); x = peak present but not quantified.

(**a**)																				
		Class	Bacillario phyceae		Chlorara chniophyceae	Chloro phyceae		Chryso phyceae		Crytophyceae				Cyano phyceae	Dinophyceae					
		Species	*P. tricornatum*	*T. weissflogii*	*C. reptans*	*C. concordia*	*D. tertiolecta*	*P. subviridis*	*R. maritime*	*C. maculata*	*C. salina*	*H brunnescens*	*R. reticulata*	*N. commune*	*A. tamarense*	*A carterae*	*G. foliaceum*	*G.galatheanum*	*G. venificum*	*H. triquetra*
Peak No.	λmax (nm)	MAA																		
1	330,346,366																			
2	292,306,320													2						
3	278			x		x		x			x		x							
4	354	Usijerene																		
5	360	Palythene													12				109	
6	314																			
7	280			x				x			x		x		x					
8	330	Asterina																		
9	310	M-glycine													2			125	310	
10	320	Palythine													5			111	503	
11	276			x		x		x		x	x		x							
12	342																	35	7	
13	294			x	x	x	x	x	x	x	x	x	x							
14	310																			
15	338	Palyth. acid													0		373	1273	553	
16	334	Porphyra-334		x											41			72	293	
17	336																		x	
18	334	Shinorine			12										1		6497	32	10	
19	352																			
20	320													2						
21	334													x						
22	336																	89	15	
23	328																			
24	384																			
25	334/336													6			44		30	
26	334																			
27	334																			
28	308					x														
29	374														x					
fg cell^−1^					12									10	61		6913	1737	1900	

(**b**)																			
		Class Species	Dino phyceae cont’d					Eugleno phyceae	Eustigmato phyceae	Prasino phyceae	Prymnesio phyceae			Raphido phyceae	Rhodo phyceae				
			*O. marina*	*P lima*	*P. micans*	*P. minimum*	*S. trochoidea*	*E. gracilis*	*N. oculata*	*N. rotunda*	*E. huxleyi*	*I. galbana*	*P. globosa*	*H. akashiwo*	*P. purpureum*	*R. marina*	*R. baltica*	Plankton 7/07/2003	Plankton 11/08/2003
Peak No.	λmax (nm)	MAA																	
1	330,346, 366		54																
2	292,306, 320																		
3	278		x									x					x		
4	354	Usijerene																	
5	360	Palythene		95	67	40	296												
6	314									x					x				
7	280			x	x			x	x	x	x			x				6	
8	330	Asterina												7					
9	310	M-glycine	37	17	149	35	176											47	24
10	320	Palythine		1		13	15											11	
11	276							x		x	x	x			x	x			
12	342																	32	4
13	294							x	x	x	x					x	x		
14	310						28										140		
15	338	Palyth. acid		x	x	12	18											28	6
16	334	Porphyra-334			314	4	5324											7	15
17	336						x											1	
18	334	Shinorine	296	8	66	31	277											41	7
19	352				1														
20	320			x	x	4	21												
21	334											x							
22	336																		
23	328					1													
24	384							x											1
25	334/336				x	5	28												
26	334				x	x													
27	334				x														2
28	308								x										
29	374													x	x				
fg cell−^1^			387	121	597	146	6155	12						7			140	173	59

**Table 2:** Origin of the cultures used in the screening study together with media used for growing and the number of cells harvested for extracting and quantifying MAAs expressed to two significant figures.

Culture	Group	a Culture code	Culture medium	Cells harvested (number * 10^6^)
*Thalassiosira weissflogii*	Bacillariophyceae	INT	f/2-Si	23
*Phaeodactylum tricornutum*	“	PLY 100	f/2-Si	9000
*Chlorarachnion reptans*	Chlorarachniophyceae	CCAP 815/1	f/2	10
*Chlamydomonas concordia*	Chlorophyceae	PLY 491	f/2	45
*Dunaliella tertiolecta*	“	PLY 83	f/2	not quantified
*Ruttnera maritima*	Chrysophyceae	PLY 177	QA	120
*Pelagococcus subviridis*	“	PLY 542	f/2	590
*Cryptomonas maculata*	Cryptophyceae	PLY 175	f/2	95
*Chroomonas salina*	“	PLY 544	f/2	210
*Rhinomonas reticulata*	“	PLY 358	Q/A	4.6
*Hemiselmis brunnescens*	“	PLY 14	Erdschreiber	17
b*Nostoc commune*	Cyanophyta	CCAP 1453/24	BG11	240
*Amphidinium carterae*	Dinophyceae	PLY 127	f/2	3.4
*Prorocentrum lima*	“	PLY 558A	f/2	99
*Prorocentrum micans*	“	INT	f/2	29
*Alexandrium tamarense*	“	PLY 173A	f/2	140
c*Oxyrrhis marina*	“	INT	f/2	4.6
*Prorocentrum minimum*	“	PLY 18B	f/2	32
*Scrippsiella trochoidea*	“	PLY 104	Erdschreiber	5.4
*Glenodinium foliaceum*	“	PLY 499	Erdschreiber	1.5
*Gymnodinium galatheanum*	“	PLY 517	Erdschreiber	0.62
*Heterocapsa triquetra*	“	PLY 169	Erdschreiber	0.05
*Gymnodinium venificum*	“	PLY 103	Erdschreiber	8
d*Euglena gracilis*	Euglenophyceae	INT	QA	150
*Nannochloropsis oculata*	Eustigmatophyceae	CCAP 849/1	f/2	125000
*Nephroselmis rotunda*	Prasinophyceae	PLY 210	QA	150
*Phaeocystis globosa*	Prymnesiophyceae	PLY 64	f/2	not quantified
*Emiliania huxleyi*	“	PLY 92	f/2	580
*Isochrysis galbana*	“	INT	f/2	1300
*Heterosigma akashiwo*	Raphidophyceae	PLY 461	f/2	380
*Porphyridinium purpureum*	Rhodophyceae	PLY 539	f/2	16
*Rhodomonas marina*	“	INT	f/2	66
*Rhodomonas baltica*	“	INT	f/2	7.6

a PLY: Plymouth Culture Collection, CCAP: Culture Collection of Algae and Protozoa, INT: sourced at PML;

b *N. commune* is a terrestrial cyanobacterium;

c Culture contained diatoms as food source;

d *E. gracilis* is a freshwater species.
